# Numerical Simulation of Fatigue Cracking of Diaphragm Notch in Orthotropic Steel Deck Model

**DOI:** 10.3390/ma16020467

**Published:** 2023-01-04

**Authors:** Yong Zeng, Hongwei He, Yu Qu, Xudong Sun, Hongmei Tan, Jianting Zhou

**Affiliations:** 1 State Key Laboratory of Mountain Bridge and Tunnel Engineering, Chongqing Jiaotong University, Chongqing 400074, China; 2 Mountain Bridge and Materials Engineering Research Center of Ministry of Education, Chongqing Jiaotong University, Chongqing 400074, China; 3 Department of Railway Engineering, Sichuan College of Architectural Technology, Deyang 618000, China

**Keywords:** OSD, diaphragm, fatigue cracking, XFEM, the first principal stress

## Abstract

Orthotropic steel deck (OSD) are widely used in steel bridges because of their many advantages, but the structures and stresses of OSD are complex and sensitive to fatigue. Based on the model test, the structural fatigue analysis of OSD is carried out by using the extended finite element method (XFEM) to understand and reveal the causes of fatigue detail cracks and the generation and propagation of fatigue cracks at the welding ends of diaphragms, U-ribs, and diaphragms, which are the main structural fatigue details of the deck. The results show that: the fatigue crack at the diaphragm opening is not caused by a single factor, but the horizontal relative displacement is the root-cause of the fatigue crack; the contribution of out-of-plane displacement to the fatigue crack is more significant than that of vertical displacement or in-plane stress, which often leads to the initiation and propagation of the fatigue crack; the crack-propagation direction is perpendicular to the contour of principal stress, and the crack propagates into the plate along the high-stress area in the horizontal direction, which is in accordance with the basic theory of crack propagation. The research methods can provide technical support for the design of similar structures.

## 1. Introduction

Orthotropic steel deck (OSD) are widely used in steel bridges because of their many advantages [1]. Yet, the structures and stresses of orthotropic steel bridge decks are complex, and there are a lot of fatigue-sensitive details [2]. The location of each fatigue detail is different, being affected by loading and deformation, and the causes of potential fatigue crack initiation and propagation are also different [3].

Scholars from all over the world have carried out a lot of research on the crack phenomenon and crack-formation process. After carrying out the fatigue test of orthotropic steel bridge deck specimens by the beach-marking method, Wang Dalei et al. [4] revealed the crack-growth process of the root-deck crack and compared the fatigue strengths for root/toe cracks. Based on local strain monitoring, Di Jin et al. [5] evaluated the fatigue performance of typical details. Liu Yang et al. [6] carried out numerical simulation on the three-dimensional mixed-mode fatigue-crack-growth behavior of an orthotropic steel-rib deck double-sided welded joint and found that the fatigue crack surface deflected during the crack-growth process, resulting in the crack finally presenting the characteristics of a space surface. The structure of orthotropic steel bridge panels is complex, with many plates and many fatigue-influencing factors. At present, there are relatively few studies on the fatigue at the openings of the cross-section of orthotropic steel bridge panels.

The reason for the fatigue crack in the diaphragm of an orthotropic deck is not clear [7]. The extended finite element method (XFEM) is seldom used in the fatigue analyses of orthotropic bridge deck structures. On the basis of the model test, it is of great significance to analyze the detailed fatigue cracks of steel bridge deck by using a powerful numerical method [8].

## 2. The Extended Finite Element Method

The XFEM, a widely used method, is a general tool for solving boundary value problems in regions with discontinuities, singularities, local deformations, and complex geometries [9].

The XFEM’s basic idea is to decompose the discontinuous displacement field into the continuous part ***u****^cont^(**X**)* and the discontinuous part ***u****^disc^(**X**)* [10]. For the elements divided by cracks, the XFEM adopts the concept of unit decomposition and introduces additional node parameters. Therefore, additional unknowns cannot be included in the element of The XFEM. The displacement discontinuity depends only on the additional node parameters. The basic principle of the XFEM is simplified to a one-dimensional crack. Although the XFEM was originally developed to study fracture problems, it has been extended to many application fields. Equation (6) of the XFEM decomposition, the most popular method for fracture analysis at present, is as follows:

***u***^*h*^(***X***) = ***u***^*cont*^(***X***) + ***u***^*disc*^(***X***) (1)

where the continuous part ***u****^cont^(**X**)* is the standard finite element interpolation; the discontinuous part ***u****^disc^(**X**)* is added according to the local unit-decomposition theorem, and the enriched information is embedded in the finite-element interpolation. The so-called enrichment is to condense the additional unknowns to the element level, so that the matrix is still sparse and the existing finite-element analysis-framework can be used to deal with the discontinuity problem.

(1) Unit decomposition of extended finite element

The concept of unit decomposition is the basis of the finite element method and the numerical manifold method, previously called the finite cover method, that was proposed earlier by Terada et al. [11]. The characteristic of the unit decomposition method is that any function *Ψ* (x) can be reconstructed by multiplying it with the unit decomposition function.

The introduction of local unit decomposition is intended to enrich the space of a numerical solution into partial differential equations. That is to say, the asymptotic solution of crack-tip field is embedded into the unit decomposition method through local unit decomposition, which greatly improves the calculation accuracy of the coarse grid. It is also possible to deal with surface effects such as nanomechanics, vacancy expansion, and interfacial diffusion.

(2) Crack and level-set method

The crack path, $\Gamma_{c}$, is described by a set of level-set functions, *f*(x), which a directed-distance function defines as follows.

(2) $$f\left(x\right)=\underset{x\in \Gamma_{c}}{\min}\|x-\bar{x}\|sign\left(n^{+}\cdot \left(\bar{x}-x\right)\right)$$

where $\bar{x}$ is the projection of point x on the discontinuous surface, $\Gamma_{\alpha }$, and n^+^ is the unit external normal vector of the discontinuity. The crack surface can be defined by a scalar function of finite element nodes and the shape function of finite elements.

**Figure 1:**
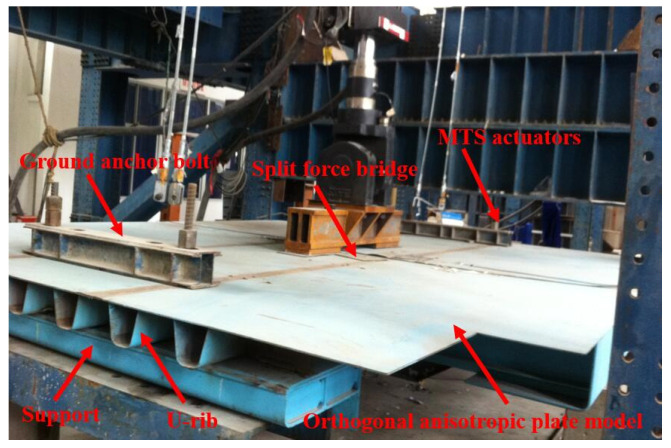
Fatigue test model.

Another set of level-set functions, *g*(x), defines the position of the crack, the origin of which is at the crack tip and the direction of which is perpendicular to the crack. Figure 1 shows the coordinate system defined by level-set function, whose r and θ are defined as follows:

(3) $$r=\sqrt{f^{2}+g^{2}},\;\theta =arctan\left(f/g\right)$$

(3) Enrichment function

Element nodes of a crack body are enriched by a directed-distance function (Equation (2)), and the enrichment function of the element nodes at the crack tip is to take the branch function as the following:

(4) $$\psi_{i=1\sim 4}=\left\{\sqrt{r}sin\frac{\theta }{2},\sqrt{r}cos\frac{\theta }{2},\sqrt{r}sin\frac{\theta }{2}sin\theta ,\sqrt{r}cos\frac{\theta }{2}sin\theta \right\}$$

As Figure 1 and Equation (4) show, r and θ are the local polar coordinates of the origin defined at the crack tip; only the first term is the bifurcation function, and the other terms are added to improve the accuracy of discontinuous problems. For the same reason, the choice of enrichment function, including the solution of the progressive displacement field at the crack tip, varies with $\sqrt{r}$. These two enrichment functions are global functions, so they have higher accuracies.

## 3. Three-Dimensional Discontinuous Extended Finite Element Model

### 3.1. Meshing

Based on the XFEM, a three-dimensional discontinuous crack growth model was established using ABAQUS software.

Meshing technology once limited the development of finite elements, and it was later found that meshless technology promoted the emergence and development of the XFEM the most. However, mesh generation plays a decisive role in the XFEM. The global coordinates and local coordinates need to be mapped to each other repeatedly. Due to the influence of calculation accuracy, a large number of algebraic iterative operations will cause error accumulation and even cause calculation failure. Therefore, a geometric algorithm was used to eliminate the influence of error accumulation. For the complex orthotropic steel deck, it was particularly difficult to mesh the components with complex structures. It can be said that the success of meshing is the key process of calculation, and three-dimensional meshing is even more difficult. Now, the three-dimensional finite element method can only be applied to planar cracks, which requires rich experience and superb meshing technology to meet the calculation requirements. For complex structures, the best method of mesh generation is to divide the components and then assemble them, but to coordinate the mesh matching among the components.

### 3.2. Numerical-Simulation Method of Crack Propagation

At present, there are two technologies to simulate crack growth: one is based on debonding (including VCCT [12]); the other is based on the viscous model.

Debonding, called node-loosening or node-releasing meaning crack propagation, can calculate a contour integral. The debonding model belongs to the damage mechanics model, which was first introduced by Barenblatt [13]. The tension-opening rule is used to simulate the unbound force of the atomic lattice so as to avoid the singularity of the crack tip.

The viscous model, or strip-buckling model, is often used to simulate thin-layer materials, crack propagation, and the interlaminar cracking of composite materials. The cohesive interface element should obey the viscous separation laws, including viscoplasticity, viscoelasticity, fracture, fiber fracture, dynamic failure, and cyclic-load failure.

Based on the XFEM, the cohesive-segment method can be used to simulate the crack description and propagation process of any path in the parent material, because the crack propagation is not bound to the element boundary. In this case, the asymptotic singularity of the crack tip need not be reflected, as only the displacement jump in the fracture element needs to be considered. Therefore, each crack propagation needs to pass through a complete element, thus avoiding the need for stress singularity in modeling.

### 3.3. Level-Set Method to Describe Geometric Discontinuity

Using extended finite element analysis, the key to simplifying the crack tracking is the geometric description of the crack, because the mesh generation need not conform to the geometric properties of the crack. The level-set method, a powerful numerical technique, can be used to analyze and calculate the interface motion in line with the requirements of the XFEM. The geometric properties of a crack can be defined by two orthogonal signed displacement functions (Figure 1). When using the traditional finite element method to calculate the path integral, it is necessary to explicitly define the crack tip and the virtual crack-propagation direction. It is usually difficult to obtain accurate path integration results on 3D surfaces, but the XFEM using the level-set method can alleviate the above defects. The singularity and geometric discontinuity can be ensured by special spread function and extra degrees of freedom. In addition, the crack tip and the virtual crack-propagation direction can be determined by the horizontal symbolic distance function.

### 3.4. Initial Crack Length

One of the limitations of the current crack-initiation life model is that it can not describe the crack size at the time of fatigue crack initiation. Therefore, the initial crack size must be assumed in the current prediction of the crack’s growth life.

The initial crack length, $a_{0}$, is the key datum for fatigue-life prediction. If the initial crack length is too small, such as 1 mm, the crack life will increase, and the risk is also high. Moreover, the applicability of the similarity principle is questionable. For such a small crack, the validity of the Paris law has not been generally supported by the empirical results. If we want to consider the small crack size, then these problems involve the uncertainty of prediction accuracy.

El Haddad et al. [14] suggested that the model should use the threshold value of a long crack-stress-intensity factor, $\Delta K_{th}$, and the virtual crack length, a, to express the fatigue limit, $\sigma_{f}$:

(5) $$\Delta K_{th}=\Delta \sigma_{f}Y\sqrt{\pi a}$$

where Y is the geometry coefficient, depending on the crack shape. For an infinite plate with a through crack whose length is 2*a*, Y=1.0. For other crack types, the value of Y can be calculated from the stress-intensity factor.

Equation (5) can be rewritten into the following equation.

(6) $$a=\frac{1}{\pi }{\left(\frac{\Delta K_{th}}{\Delta \sigma_{f}Y}\right)}^{2}$$

Equation (6) is the recommended equivalent initial crack length (EIFS). If the initial crack length of the specimen is EIFS and is less than the fatigue-limit stress amplitude, the fatigue life calculated by the fracture mechanics method is infinite, meaning that the fatigue life is lower than the fatigue limit. The fatigue limit is equivalent to the fatigue strength of 10^7^~10^8^ cycles.

The main problem of life prediction based on fracture mechanics method is in determining the initial crack size.

Hobbacher [15] considered that the threshold value of stress intensity factor for C and CM steel structures are the following.

(7) $$K_{th}=\{\begin{array}{c} 6\;\mathrm{MPa}\sqrt{m}\;\;\;\;R=0\\ 6.0-4.6r\;\;\;\;\;\;R>0 \end{array}$$

It can be seen that the threshold value of the maximum stress intensity factor is 6 MPa.

For Q345qD bridge steel, if the stress ratio of this test (R=0.2215) is used for calculation, we can obtain $\Delta K_{th}=4.981\;\mathrm{MPa}\sqrt{m}$.

According to the literature [16], the $\Delta K_{th}$ values of bridge steel Q370qE are as follows.

(8) $$\Delta K_{th}=5.556{\left(1-0.825R\right)}^{1.147}$$

where *R* is the loading-stress ratio. According to the stress ratio in this paper, it is obtained that $\Delta K_{th}=4.408\;\mathrm{MPa}\sqrt{m}$.

The $\Delta K_{th}$ value of Q345qD is $63N\cdot {mm}^{-\frac{3}{2}}=2.032\;\mathrm{MPa}\sqrt{m}$, which is equivalent to the stress ratio *R* = 0.76. In this paper, the constant amplitude fatigue limit of the U-rib flat-steel box girder is 70 MPa [17]. According to Equation (6), the initial crack length is 1.262 mm, which is regarded as the initial crack length of crack propagation in this paper.

It can be seen that the initial crack size is closely related to the fatigue limit, stress ratio of the materials, and fatigue details. For example, the initial crack size of aluminum alloy cannot be taken as the initial crack size of bridge steel. Firstly, the ductility of bridge steel is higher than that of aluminum alloy, and the isotropic properties of aluminum are better than those of bridge steel. Secondly, different fatigue details have different constant amplitude fatigue limits, so the initial crack sizes are different.

The practical method involves the use of experience to assume the initial crack length, such as metal being assumed to be 1~3 mm. Another method is to use the results of non-destructive testing (NDI). In short, as long as there is no major damage to the structure, even if using a too-small initial crack length to evaluate the crack life is risky, the final crack length has little effect on its life.

### 3.5. Crack-Initiation Criterion and Propagation Direction

The maximum principal stress criterion can be expressed as the following:

(9) $$f=\frac{\langle \sigma_{max}\langle \rangle \rangle }{\sigma_{max}^{0}}$$

where $\sigma_{max}^{0}$ is the critical maximum principal stress. The symbol <∙> represents the Macaulay bracket. The bracket is used to indicate that pure compressive stress does not cause initial damage. When the maximum stress ratio reaches a certain value, the damage begins. The direction of propagation is the direction of maximum shear stress.

### 3.6. Material Properties

In the finite element analysis, it is assumed that the material is a 14MnNbq bridge steel plate with ideal linear elasticity, the elastic modulus E is 2.1 × 10^5^ MPa, the yield strength is not less than 370 MPa, the fracture strength is not less than 530 MPa, the Poisson’s ratio is 0.3, and the V-type impact energy is not less than 41 J. In this paper, the failure criterion based on the evolution of damage mechanics is adopted. The damage criterion is the maximum principal stress, and the failure criterion is the damage-initiation criterion. The damage-evolution coefficient is 5 × 10^−5^, and the normal fracture energy is 42.2 kN/m. The impact-toughness of Q345qD bridge steel is 34 J. The constant amplitude fatigue limits of different fatigue details of orthotropic steel bridge decks are different. The cracks appear at the hole of the diaphragm, and the constant-amplitude fatigue limit of fatigue details is 70 MPa.

## 4. Extended Finite Element Model of OSD from Experimental Model

**Figure 2:**
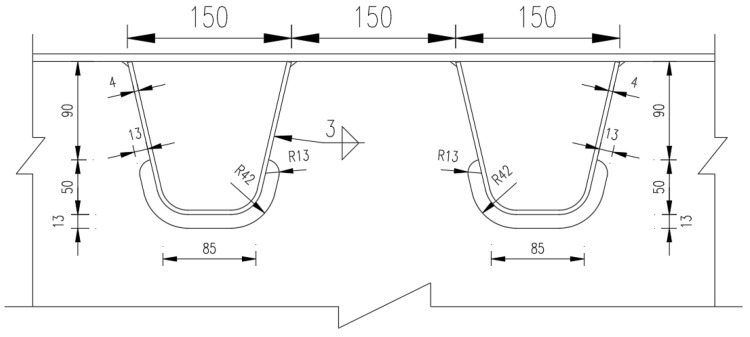
Geometry of U-rib and cutout details in fatigue model (in mm).

**Figure 3:**
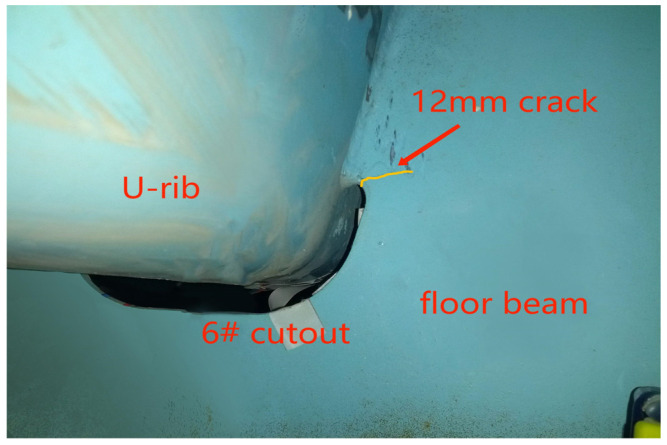
Cutout fatigue crack in the fatigue test model.

The supporting project was a suspension bridge. In the design process of the steel-deck model, the supporting project was simulated according to the elastic-support continuous beam [18]. After the overall analysis, the steel box girder with 16 m standard section was selected and analyzed by the elastic-simple-support finite element method. In the light of the analysis results of the segmental-steel box girder and the local characteristics of the orthotropic-plate fatigue details which are only sensitive to wheel load, a simplified panel segment model was established on the scale of 1:2, shown in Figure 1 and Figure 2. As the above analysis shows, the boundary condition of the panel model was set as simply supported at both ends of the longitudinal bridge. One end constrained three translational degrees of freedom of the nodes, the other end only constrained the vertical displacement of the nodes, and the transverse bridge only restricted the downward-displacement on both sides. To summarize, the calculation results were in good agreement with the original bridge type. A cutout fatigue crack was found in the fatigue test model, as shown by the yellow wavy line in Figure 3. The crack’s study is outlined below.

**Figure 4:**
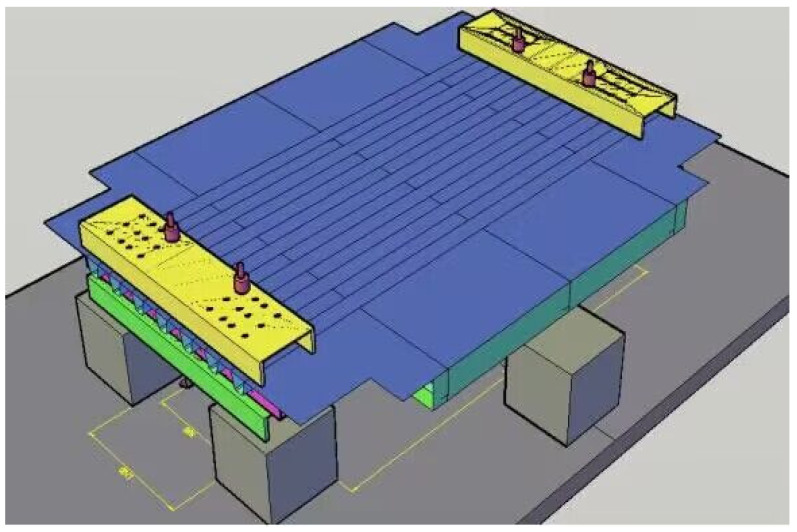
The model of OSD segmental deck.

The discontinuous three-dimensional extended finite element model of orthotropic steel bridge deck was established by ABAQUS software. Both ends of the U-rib along the bridge direction were simply supported vertically, and both sides of the diaphragm along the bridge direction were only limited to the downward displacement. The numerical model was divided into 190,791 C3D8R units and 383,344 nodes. According to the average value of the relative displacement along the bridge between the horizontal displacement measuring points and the roof plate measured in the test, there were two working conditions of relative displacement and no relative displacement in the corresponding position of the model, and the vertical load was 62.7 kN, which were all calculated by the XFEM. The overall model of the bridge segmental deck is shown in Figure 4.

The crack propagation was calculated by the discontinuous Galerkin propagation finite element method (DG-XFEM). The boundary conditions of the numerical model were consistent with the experimental model. The deck model was simply supported along the model to avoid unexpected rigid-body movement. Both sides were placed on the concrete pier to limit the downward displacement.

The crack type, propagation region, and propagation path were set as a through crack, the whole diaphragm, and any path, respectively. Due to the facts that the calculation of a crack growth is actually a large deformation problem and the crack propagation itself is a strong discontinuous problem, geometric nonlinearity was used in the analysis process, as it would lead to the non-convergence of the iteration of the solution process. In addition, the incremental step of the solution would be very small, which would lead to a long solution time. The initial crack length was 1.26 mm.

## 5. Fatigue Cracking Analysis of OSD Using XFEM

### 5.1. Cause-Analysis of Fatigue Crack at the Joint-of-Arc Opening, U-Rib, and Diaphragm

**Figure 5:**
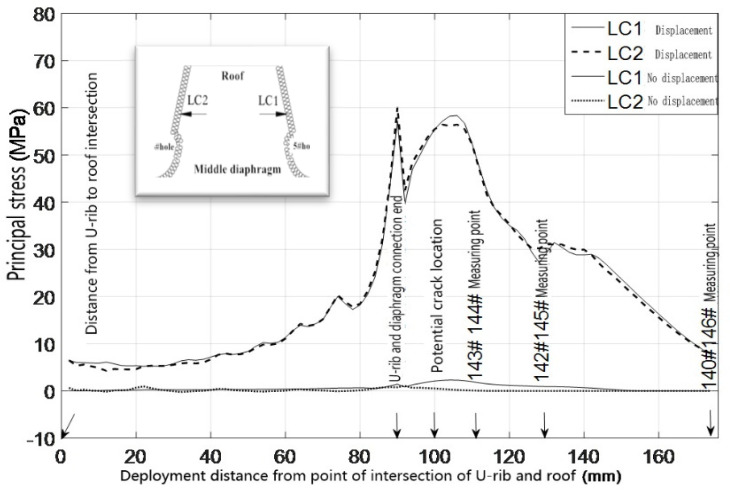
Principal stress distribution around the left side of 4# hole and the right side of 5# hole in diaphragm.

**Figure 6:**
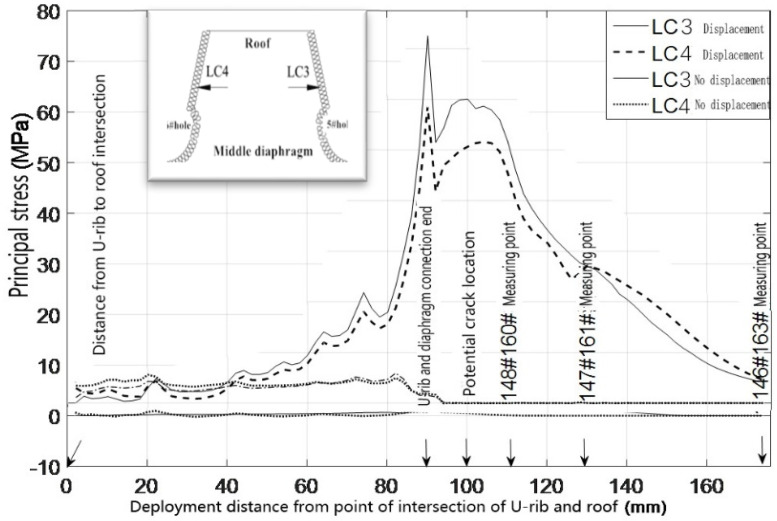
Principal stress distribution around the left side of the diaphragm 5# hole and the right side of the diaphragm 6# hole. Note: LC1: 4# hole left peripheral unit; LC2: 5# hole right peripheral unit; LC3: 5# hole left peripheral unit; LC4: 6# hole right peripheral unit.

The cracks appeared on the right side of the sixth hole opening and near the end of the welding seam between the U-rib and diaphragm. The relationships between the principal stress of the peripheral elements on the left side of the fourth hole, the left and right sides of the fifth hole, and the right side of the sixth hole, and the distance from the roof to the expansion, are drawn in Figure 5 and Figure 6, respectively.

What can be seen from Figure 3 and Figure 4 is as follows.

(1) When there is no horizontal relative displacement between the top plate and diaphragm, the principal stress along the left side of the fourth hole, the left and right side of the fifth hole, and the right side of the sixth hole is very low (as shown in the upper right figure), and there is no stress-concentration at the welding-end of the U-rib and diaphragm. The maximum principal tensile stress on the right side of the sixth hole is not more than 7 MPa. Even if the hot-spot stress is considered, it is far from the constant-amplitude fatigue limit of the fatigue detail. In other words, the crack will not occur.

(2) The maximum principal stress is 60.6 MPa and the hot-spot stress is 74.2 MPa at the minimum cross section of the diaphragm opening, both of which exceed the constant-amplitude fatigue limit of the fatiguing detail, which indicates that the fatigue details have the necessary conditions for crack initiation. At the end of the welding seam between the U-rib and the diaphragm, an obvious stress concentration appears, and the amplitude is close to or exceeds the constant-amplitude fatigue limit of the fatigue detail. Considering the hot-spot stress, the amplitude of the principal stress exceeds the constant-amplitude fatigue limit of the fatigue detail, which shows that the horizontal relative displacement is the fundamental cause of the crack at the diaphragm opening, and that the hot-spot stress and structural defects promote the crack heavily.

To summarize, the fatigue cracking first appears in the fatigue-vulnerable part, which is determined by the fatigue stress amplitude, the number of actions, and the resistance of structural details, and then expands when enough energy can be provided around it, which is why the control part is used to determine the fatigue performance of an orthotropic steel deck. There are geometric discontinuities, local stress concentrations, hot-spot stresses, and welding defects at the opening of the diaphragm and the end of the connecting weld between the U-rib and diaphragm. They are fatigue-sensitive parts, which are greatly affected by the relative horizontal displacement. They are high-incidence areas of fatigue cracks, with occurrence rates as high as one/three or more. They are the control parts of fatigue performance of orthotropic steel deck [19].

### 5.2. Fracture-Mechanics Analysis of Crack Propagation

**Figure 7:**
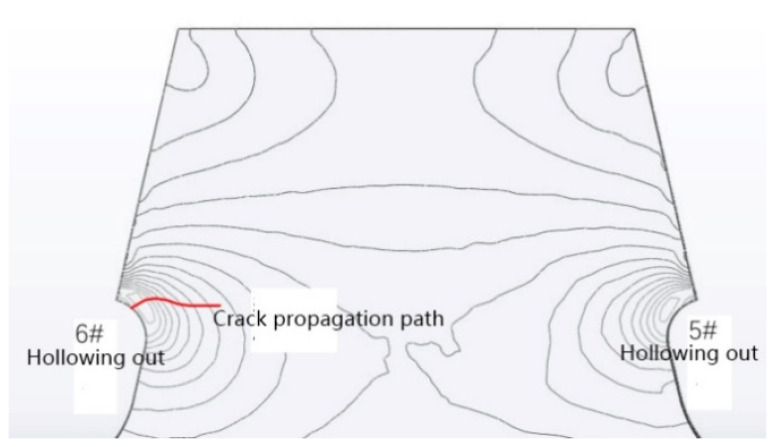
Contour of principal stress and crack-propagation path on the right side of 6# arc opening.

The location of the crack was determined according to the test, and the crack propagation was calculated by the DG-XFEM. The initial crack length was taken as 1.26 mm, and the principal stress contour and crack-propagation path of the fifth and the sixth holes without cracks are shown by the red wavy line in Figure 7. The simulated crack length was 32.37 mm, which was 31 mm measured in the test, and the difference between them was very small.

**Figure 8:**
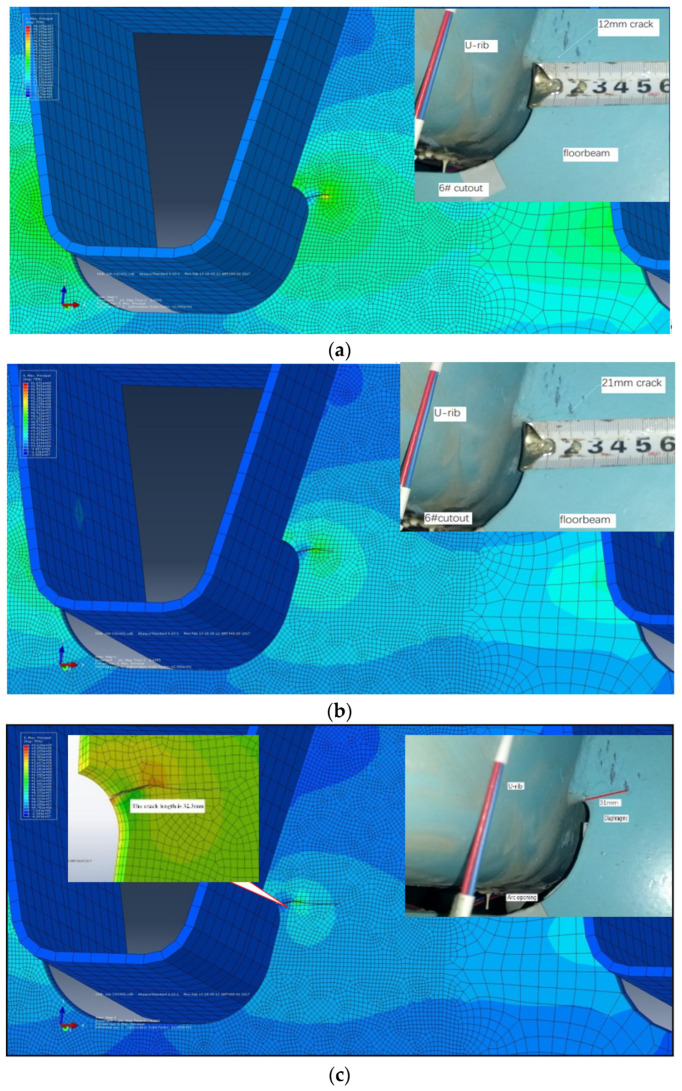
Comparison of simulated crack-propagation and test photos, the top left is the test crack. (**a**) Simulation and experimental comparison of 12 mm crack; (**b**) simulation and experimental comparison of 21 mm crack; (**c**) simulation and experimental comparison of 31 mm crack.

Figure 8 shows the comparison of the crack growth lengths of 12 mm, 22 mm, and 31 mm in the model test under 2.2 million times, 2.4 million times, and 2.6 million times loading.

It can be seen that the crack begins to propagate along the direction perpendicular to the maximum principal stress, which conforms to the basic theory of crack propagation. The stress field at the crack tip changes with the crack propagation, and the crack-propagation direction changes slightly and tends to become horizontal gradually. The numerical simulation results and the crack location and morphology are consistent with the test results and the results of the real bridge, respectively [20,21], and the cracks in the diaphragm of a real bridge are basically consistent with those [22].

### 5.3. Fatigue Analysis of the Cross Joint of the Roof Plate, Diaphragm, and U-Rib

**Figure 9:**
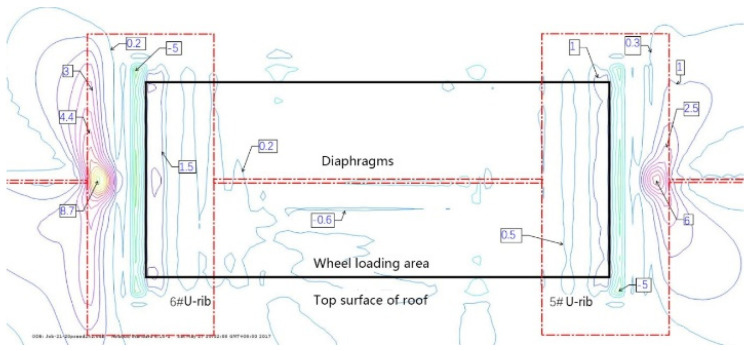
Contour of the principal stress on top surface of roof plate at wheel trace (MPa).

**Figure 10:**
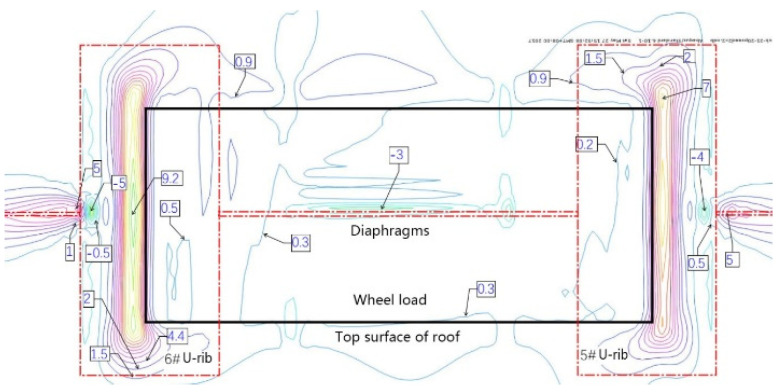
Contour of the principal stress of roof bottom at wheel trace (MPa).

The intersection of the roof, diaphragm, and U-rib is an important structural detail of an orthotropic steel deck. According to the orthotropic steel-deck model, the fatigue stress is calculated. Figure 7 and Figure 8 show the contour maps of the principal stresses on the upper and lower surfaces of the top plate at the wheel trace, and Figure 9 and Figure 10 also show the contour maps of the principal stresses on the elevation of the connection between the roof plate and the diaphragm plate near the wheel trace.

**Figure 11:**
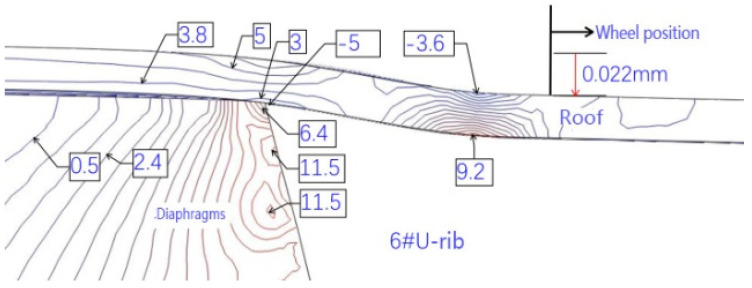
Principal-stress contour of the vertical plane at the junction of roof, U-rib, and diaphragm.

The fatigue crack in the roof plate is always related to the connection among the roof, U-rib, and diaphragm [23,24]. The contour lines of the upper and lower surfaces of the roof show that the upper and lower surfaces of the roof are compressive stresses within the range of the wheel load. Outside of the wheel trace, the principal stress at the top of the roof is −5 MPa, which indicates that the roof is mainly under pressure, and the principal stress at the bottom of the corresponding roof is 9.2 MPa, which indicates that the roof is mainly under tension. At the junction of the diaphragm and the sixth U-rib, the main stress at the top of the roof is about 9 MPa, which means that it is mainly in tension. At the bottom of the corresponding roof, the main stress is −5 MPa, which indicates that it is mainly in compression. This shows that the roof between the sixth U-rib and the wheel trace is in a complex repeated-bending state, and the principal stress amplitude is about 14 MPa. Although it is not high, the compressive stress is so high that the displacement amplitude is about 0.022 mm (seen in Figure 11). From the above analysis, it can be seen that the fatigue-resistant ability of the plate is weak, and the plate is sensitive to external deformation. Hence, enough attention should be paid to the fatigue problem. Due to the fact that there are cracks in the roof, rainwater will be immersed into the U-rib, resulting in corrosion and aggravating fatigue. Such cracks are difficult to find. Once found, it is too late.

At the junction of the top plate, U-rib, and diaphragm, an obvious stress concentration appears in the top plate. In the previous design, there were welding holes in the diaphragm which would obviously aggravate the stress concentration and induce fatigue cracks. Now that the welding hole has been canceled, the cracks disappear. Through continuous improvement of the design, such as multiple weldings between the U-rib and top plate, the welding quality is improved, the weld size is increased, the stress-state of top plate is improved, and the probability of crack-occurrence is reduced. In recent years, the crack was obviously reduced, and it is no longer the dangerous control part of fatigue cracking.

**Figure 12:**
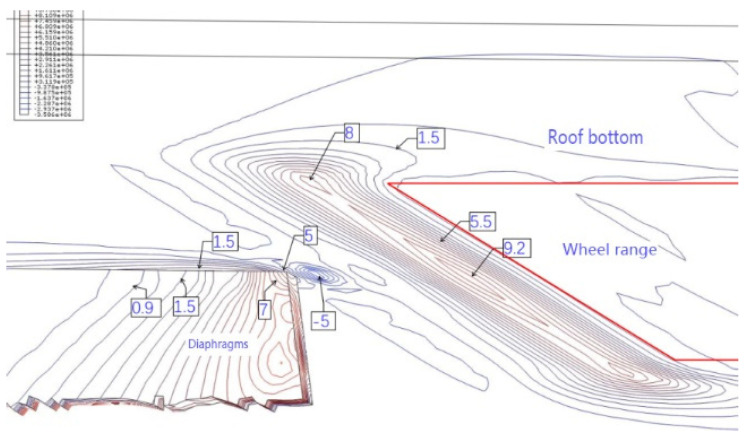
Isoline of 3-D principal stress at the joint of roof, U-rib, and diaphragm (U-rib not shown) (MPa).

It is worth noting that the tensile stress at the connection between the top plate and the diaphragm on the left side of the sixth U-rib is about 5 MPa, and the maximum stress amplitude is about 10 MPa. This shows that with the wheel-load moving along the transverse position, the stress state between the top plate and the diaphragm will change from the compression cycle to the tension compression cycle. It can be seen more clearly from Figure 12 that in the current situation, the left diaphragm and the top plate will have tensile stress, and with the wheel load moving laterally, all of the compressive stress states will appear. This is consistent with the test results.

## 6. Conclusions

A three-dimensional discontinuous propagation finite element model of OSD from the experimental model is established based on the XFEM, and the generation and propagation of fatigue cracks at the welding ends of diaphragms, U-ribs, and diaphragms, which are the main structural fatigue details of the deck, are studied by the XFEM. Conclusions can be drawn in the following.

1. The result of the XFEM analysis shows that under the condition of 1.21 mm horizontal relative displacement between the diaphragm and the top plate, the cracks appear in the area with the maximum principal tensile stress at the opening of the diaphragm, and the stress is as high as 60.6 MPa. Considering the unfavorable factors such as stress concentration, hot-spot stress, and welding defects of the fatiguing detail, the main tensile stress can reach or exceed the constant amplitude fatigue limit of 70 MPa, which promotes the crack.

2. The horizontal relative displacement is the root cause of the fatigue crack, the fatigue crack at the diaphragm opening is not caused by a single factor, and structural defects promote the cracking.

3. The contribution of out-of-plane displacement to fatigue cracks is more significant than that of vertical displacement or in-plane stress, which often leads to the initiation and propagation of fatigue cracks in the diaphragm. The diaphragm is an important part of the panel structure, and the upper area mainly transmits pressure. If only vertical displacement or in-plane stress is used for fatigue assessment, it may lead to misjudgments.

4. The analysis of crack propagation by the DG-XFEM shows that the crack-propagation direction is perpendicular to the contour of the principal stresses, which is consistent with the basic theory of crack propagation. The crack propagates into the plate along the high-stress area in the horizontal direction. With the decrease of stress intensity, the crack propagation stops gradually. The crack-propagation path is consistent with the experimental and real bridge results, which shows that the analysis in this paper is reasonable.

5. The equivalent initial crack length is calculated and discussed by using the threshold values of the stress-intensity factor of bridge steel, which provides a basis for estimating crack-propagation life.

## Data Availability

All data generated or analyzed during this study are included in this article. All data included in this study are available upon request by contact with the corresponding author.
